# Comparison of a novel tablet formulation of tacrolimus and conventional capsule formulation in *de novo* kidney transplant recipients: a systematic review and meta-analysis

**DOI:** 10.3389/fphar.2023.1310339

**Published:** 2023-12-08

**Authors:** Zhenyu Liu, Kexin Yin, Huiqian Liu, Ning Wang, Junjie Yao, Jiangtao Zhou, Yongxi Tang, Zhikang Yin

**Affiliations:** ^1^ Department of Urology, The First Affiliated Hospital of Chongqing Medical University, Chongqing, China; ^2^ The First Clinical College of Chongqing Medical University, Chongqing, China; ^3^ Department of Urology, The First People’s Hospital of Chongqing Liang Jiang New Area, Chongqing, China

**Keywords:** kidney transplantation, relative bioavailability, pharmacokinetic, tacrolimus, tablet

## Abstract

**Background:** The work aimed to compare the pharmacokinetic (PK) profiles and other outcomes reported in observational studies in *de novo* kidney transplant recipients (KTRs) receiving novel once-daily extended-release tablet tacrolimus (LCPT; LCP-tacrolimus; Envarsus XR) or receiving standard-of-care capsule tacrolimus (PR-Tac; prolonged-release tacrolimus; Advagraf/IR-Tac; immediate-release tacrolimus; Prograf).

**Methods:** A systematic review was conducted for all randomized controlled trials (RCTs) and cohort studies investigating the outcomes in KTRs receiving LCPT or PR-Tac/IR-Tac. We systematically searched PubMed, Web of Science, and EMBASE, with no language restriction. The registered trials and references listed in relevant studies were also searched. Data were extracted for the PK profile, tacrolimus trough level (TTL), and changes in the estimated glomerular filtration rate (eGFR) and serum creatinine (Scr), biopsy-proven acute rejection (BPAR) rate, delayed graft function (DGF) rate, post-transplant diabetes mellitus (PTDM) rate, tremor rate (TR), death rate (DR), and rate of infection by cytomegalovirus (CMV). This study was registered with PROSPERO (registration number: CRD42023403787).

**Results:** A total of seven eligible articles including 1,428 patients with 712 in the LCPT group versus 716 in the PR-Tac/IR-Tac group were included in this study for evidence synthesis. The baseline characteristics of the LCPT, PR-Tac, and IR-Tac groups were similar. The pooled analysis showed a higher PK profile in the LCPT group, and this result was consistent with those of all the included studies. In addition, no significant difference was observed for other outcomes.

**Conclusion:** Considering heterogeneity between studies and potential bias, care providers should select agents based on patient-specific factors and their clinical experience for the immunosuppressive treatment of *de novo* KTRs.

## 1 Introduction

Tacrolimus was discovered in 1984 and introduced into clinical use soon after, making an outstanding contribution to the success of solid organ transplants worldwide (Ong and Gaston, 2021). In recent years, studies have also shown that tacrolimus can be used in the treatment of lupus nephritis (Zheng et al., 2022), vitiligo (Ebrahim et al., 2021), adults with *de novo* minimal change disease (Medjeral-Thomas et al., 2020), severe facial seborrheic dermatitis (Joly et al., 2021), refractory posterior blepharitis (Sakassegawa-Naves et al., 2017), myasthenia gravis (Bao et al., 2019), kaposiform hemangioendothelioma and tufted angioma, resistant ulcerative proctitis (Lawrance et al., 2017), etc.

Tacrolimus is a powerful immunosuppressive agent that inhibits the activation and proliferation of T cells and the response of B cells dependent on T cells, and is also a kind of calcineurin inhibitor (CNI) (Czarnecka et al., 2023). CNI has been widely selected for preventing acute rejection after renal transplantation (Veenhof et al., 2020). Because of the restrictive therapeutic index (Ko et al., 2021), maintaining an appropriate concentration of tacrolimus levels in the blood is critical for the prevention of organ rejection and minimization of toxicity after renal transplantation that requires individual dose adjustment and close drug monitoring (Fernandez Rivera et al., 2022). Both inadequate and excessive doses of tacrolimus may have an impact on the outcomes because they expose patients to the risk of graft rejection or adverse events related to immunosuppression treatments (Kamar et al., 2019). Tacrolimus is available in three different dosage forms (Vadcharavivad et al., 2019). Two of the three dosage forms have divergent mechanisms of release and are of prolonged-release, once-daily formulation, with one form using MR-4 formulation technology (PR-Tac) and the other using MeltDose formulation technology (LCPT). The remaining one dosage form is of immediate-release, twice-daily formulation (Czarnecka et al., 2023). Referring to the previously published literature, the tablet formulation in this study was given to the LCPT group and the capsule formulation was given to the IR-Tac/PR-Tac group (Kim et al., 2017).

Some studies have indicated that these tacrolimus formulations have different pharmacokinetic profiles and require different dosages to achieve similar blood levels (Jennifer Trofe-Clark et al., 2017; Vadcharavivad et al., 2019). Usually, a higher dosage is required for PR-Tac than IR-Tac to maintain similar tacrolimus trough levels (TTLs). However, LCPT requires less dosage than IR-Tac (Banas et al., 2020). Prolonged-release tacrolimus has lower variability in intra-patient exposure to improve patient compliance and convenience after kidney transplantation compared with immediate-release tacrolimus (Kim et al., 2017). These superior characteristics of MR-4 ER-Tac (PR-Tac) and MeltDose ER-Tac (LCPT) have generated significant interest as a high variability in intra-patient exposure to tacrolimus and non-adherence to tacrolimus therapy has an association with poor outcomes for a long-term transplant (Borra et al., 2010).

## 2 Methods

### 2.1 Literature search

This analysis based on evidence was performed according to the Preferred Reporting Items for Systematic Reviews and Meta-Analyses (PRISMA) statement and was prospectively registered with PROSPERO (CRD42023403787). PubMed, Web of Science, and EMBASE were systematically searched from the inception of each database to 6 March 2023 for potentially eligible studies. No restrictions were imposed on language. Terms used in the search included kidney transplantation, kidney transplantations, renal transplantation, renal transplantations, renal grafting, kidney grafting, LCPT-tacrolimus, Envarsus-tacrolimus, LCP-tacrolimus, Prograf, and Advagraf. In addition, a manual review of references in all studies which met the eligibility criteria was conducted. Two investigators separately located and assessed the incorporated studies. Any disagreements that arose in the process of the literature search were resolved through negotiation with a third researcher.

### 2.2 Identification of qualified studies

The retrieved studies were included in this study if they met the following criteria (Ong and Gaston, 2021): the study design belonged to randomized controlled or cohort studies (Zheng et al., 2022); studies on *de novo* kidney transplant recipients (KTRs) with recipients who were re-transplanted, reporting outcomes after receiving tablet formulation (LCPT) and including capsule formulation (PR-Tac/IR-Tac) as the control group (Ebrahim et al., 2021); one or more of the following outcomes were assessed: pharmacokinetic (PK) profile, TTL, and changes in eGFR and Scr, biopsy-proven acute rejection (BPAR) rate, delayed graft function (DGF) rate, post-transplant diabetes mellitus (PTDM) rate, tremor rate (TR), death rate (DR), and rate of infection by CMV (Medjeral-Thomas et al., 2020); sufficient data were available to calculate odds ratios (ORs) or the weighted mean difference (WMD).

Reviews, letters, editorial comments, conference abstracts, case reports, articles on pediatrics, and non-published articles were excluded.

### 2.3 Data extraction

Two researchers separately performed data extraction. A third researcher made the ultimate decision on any discrepancy that arose in the process. Data from the included studies were extracted, including the first author, study period, country of study, publication year, study design, sample size, age, gender, body mass index (BMI), diabetes at the time of transplant, cold ischemic time (CIT), race, donor typing, panel reactive antibodies, previous transplant, pharmacokinetic profile, TTL, changes in eGFR and Scr, BPAR rate, and other outcomes. If the data on the same population were reported simultaneously in different studies, the data from the most recent studies were collected. When continuous variables expressed as the mean value with a range or interquartile range appeared in the study, they were converted to the mean ± standard deviation using a validated mathematical method (Wan et al., 2014; Luo et al., 2018). If data were not available or not reported in a study, the proper author was contacted for complete information.

### 2.4 Quality assessment

The quality of the cohort studies included in the current study was assessed using the Newcastle–Ottawa Scale (NOS) (GA Wells et al., 2011), and a score of 7–9 indicated high quality (Kim et al., 2019). In addition, the quality of RCT was evaluated using the Cochrane Handbook 5.1.0 risk scale for bias. The evaluation covered these aspects: blinding of participants and personnel, random sequence generation, blinding of the outcome assessment, allocation concealment, selective reporting, incomplete outcome data, and other sources of bias, and each was assessed with a low risk, high risk, or unclear risk. More “low-risk” bias evaluations indicated higher quality. Two investigators separately assessed the quality and the evidence level of the included studies, and all disagreements were addressed by discussion with a third researcher.

### 2.5 Statistical analysis

Evidence synthesis was implemented using Review Manager version 5.4 (Cochrane Collaboration, Oxford, United Kingdom). The WMD was used for the comparison of continuous variables. The OR was used for the comparison of dichotomous variables. All indicators were subject to a 95% confidence interval (CI) when reported. The chi-squared (χ^2^) test (Cochrane^’^s Q) and inconsistency index (I^2^) were used to evaluate heterogeneity between studies (Higgins and Thompson, 2002). The *p*-value from χ^2^ test <0.05 or I^2^ > 50% indicated remarkable heterogeneity. For significant heterogeneity, a random-effects model was used to evaluate the pooled WMD or OR (*p*-value from the χ^2^ test <0.05 or I^2^ > 50%). Otherwise, a fixed-effects model was used. In addition, a one-way sensitivity analysis was conducted to assess the influence of the included studies on joint outcomes with significant heterogeneity. Review Manager 5.3 version (Cochrane Collaboration, Oxford, United Kingdom) was used to create funnel plots to visualize the assessment results of publication bias.

## 3 Results

### 3.1 Literature search and characteristics of studies

**FIGURE 1:**
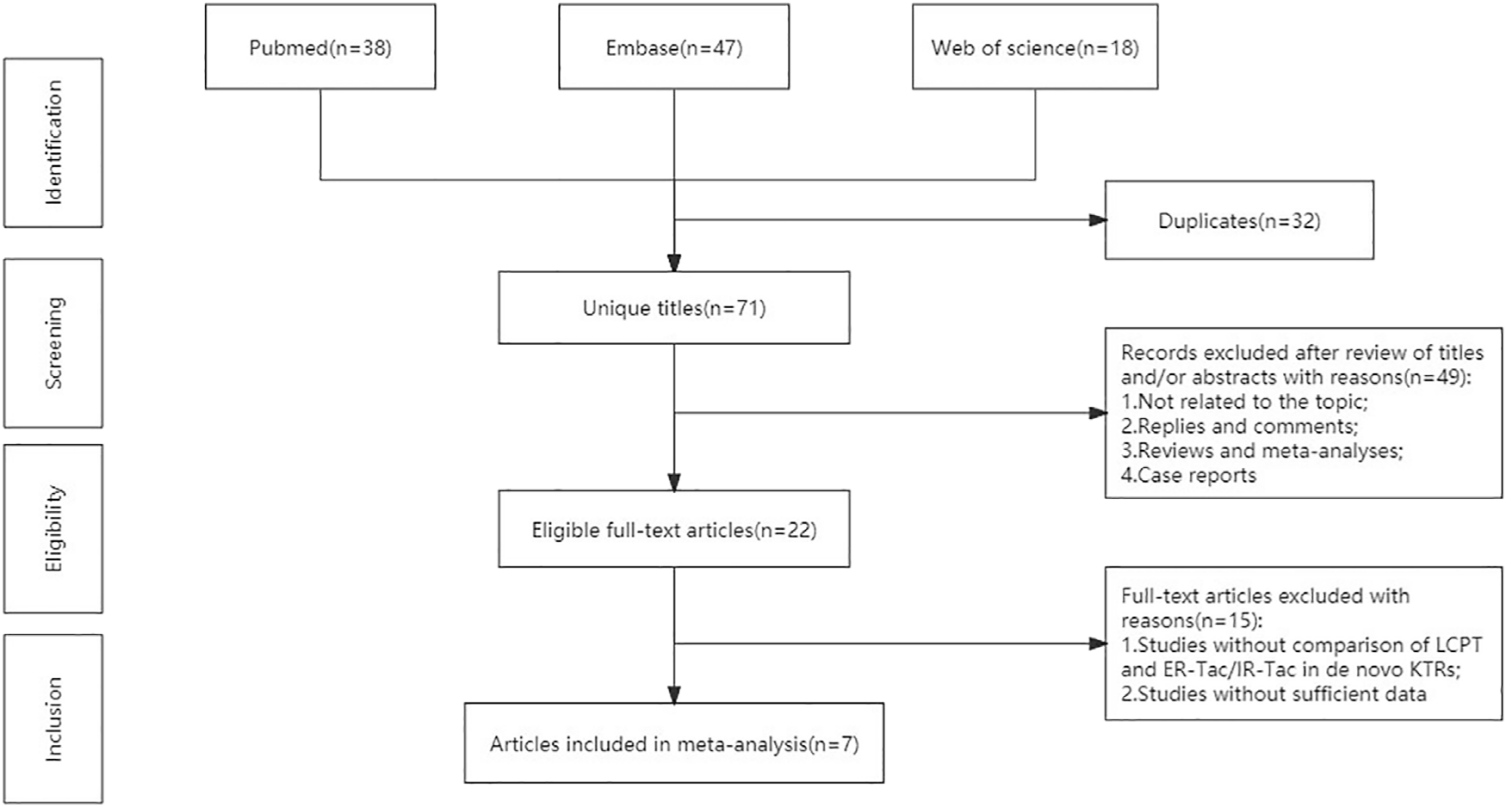
Flowchart of the systematic search and selection procedure.

**FIGURE 2:**
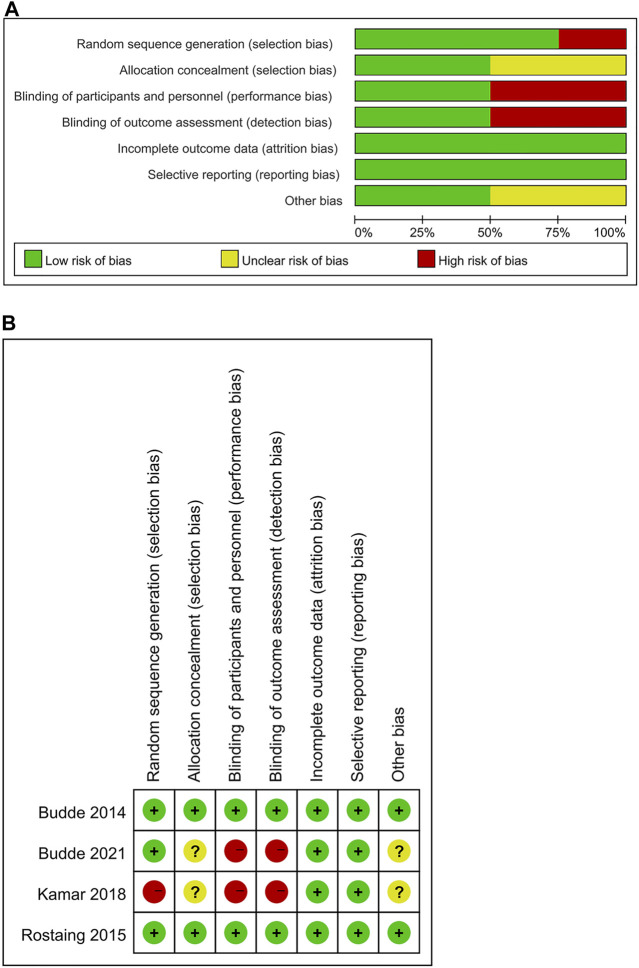
Bias evaluation chart of RCT. **(A)** Risk of bias graph. **(B)** Risk of bias summary.

**FIGURE 3:**
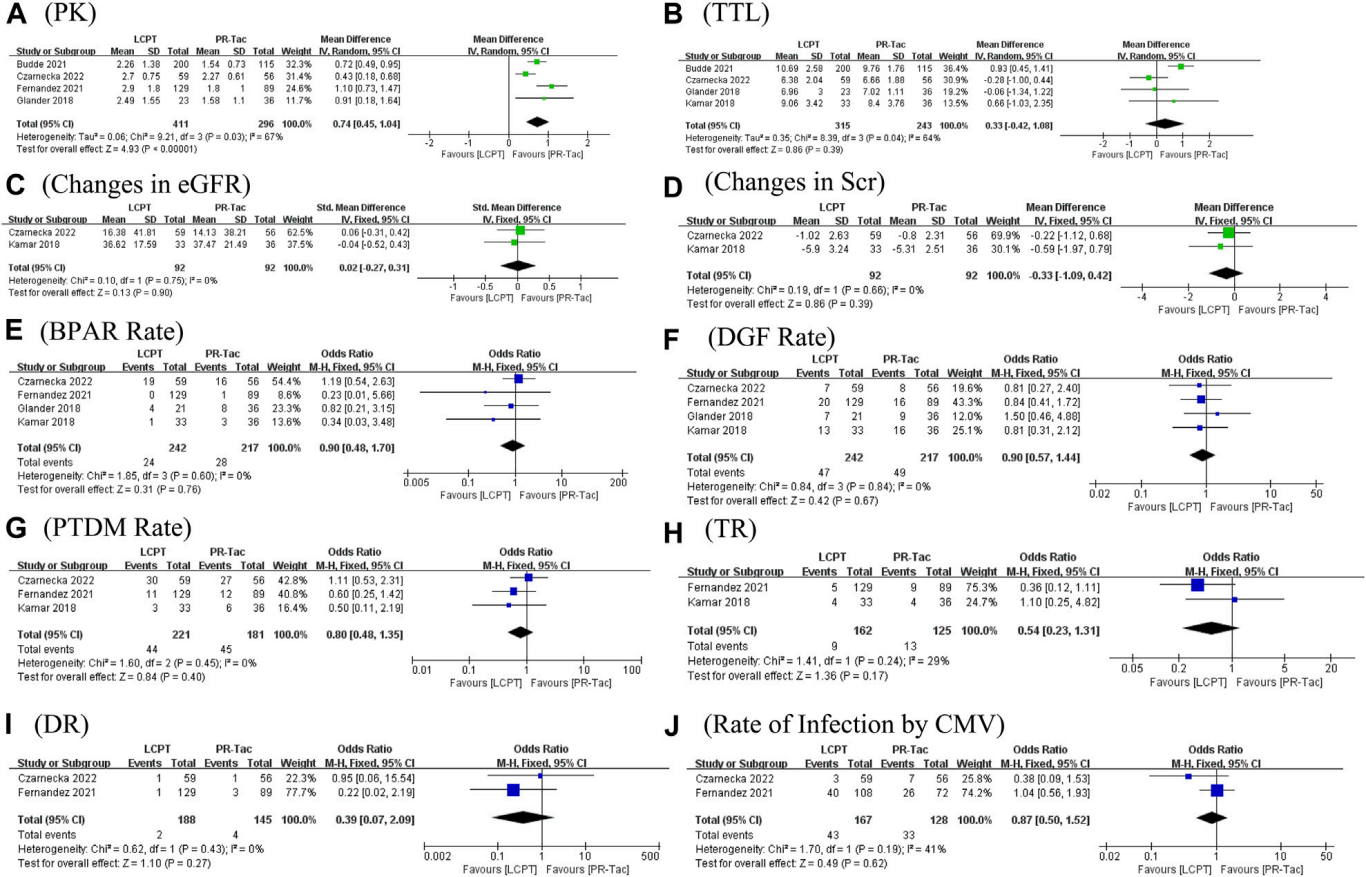
Forest plots of outcomes. **(A)** PK; **(B)** tacrolimus trough level; **(C)** changes in eGFR; **(D)** changes in Scr; **(E)** BRAR; **(F)** DGF rate; **(G)** PTDM rate; **(H)** TR; **(I)** DR; and **(J)** rate of infection by CMV.

**FIGURE 4:**
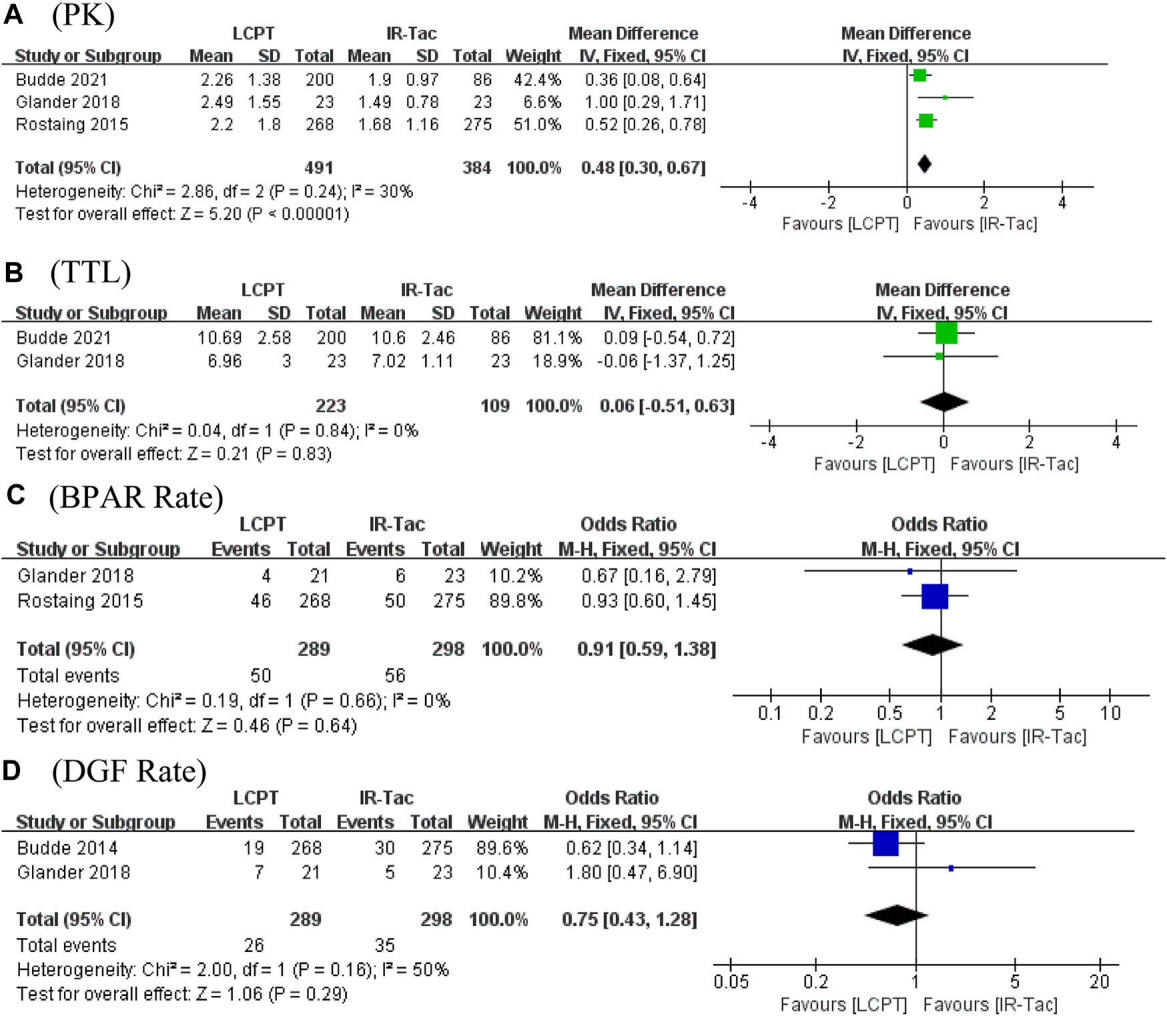
Forest plots of **(A)** PK profile; **(B)** tacrolimus trough level; **(C)** BPAR rate; and **(D)** DGF rate.

**FIGURE 5:**
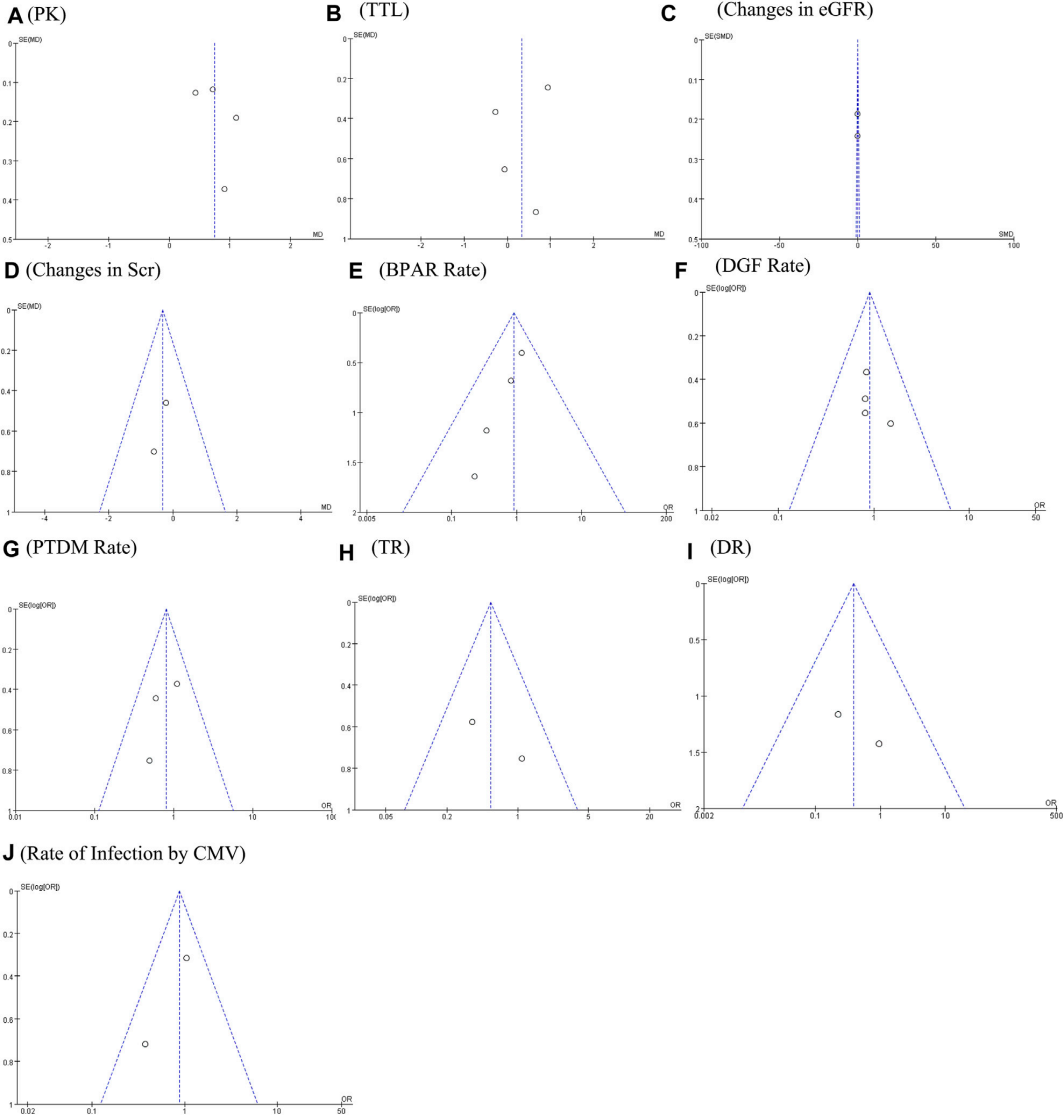
Funnel plots of **(A)** PK profile; **(B)** tacrolimus trough level; **(C)** changes in eGFR; **(D)** changes in Scr; **(E)** BRAR; **(F)** DGF rate; **(G)** PTDM rate; **(H)** TR; **(I)** DR; and **(J)** rate of infection by CMV.

**FIGURE 6:**
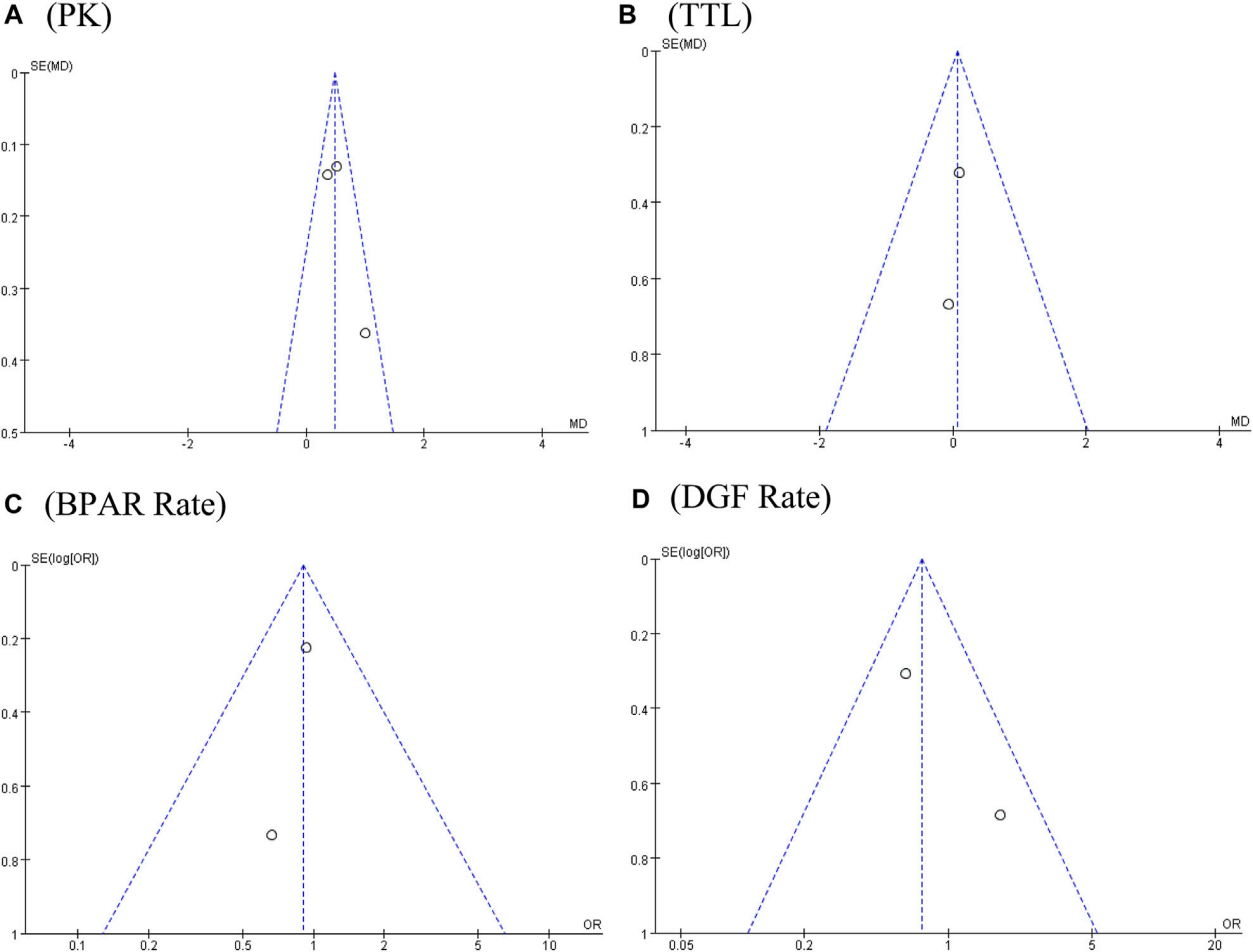
Funnel plots of **(A)** PK profile; **(B)** tacrolimus trough level; **(C)** BPAR rate; and **(D)** DGF rate.

The flowchart displays the systematic search and study selection procedure (Figure 1). A total of 103 articles were identified through the systematic literature search, including 38 from PubMed, 47 from EMBASE, and 18 from Web of Science. After the duplicates were excluded, the titles and abstracts of 71 papers were reviewed. Ultimately, seven articles met the criteria, and a total of 1,971 patients (980 in the LCPT group versus 991 in the PR-Tac/IR-Tac group) from these articles were included in the meta-analysis, including three cohort studies (Glander et al., 2018; Fernandez Rivera et al., 2022; Czarnecka et al., 2023) and four randomized studies (Budde et al., 2014; Rostaing et al., 2016; Kamar et al., 2019; Budde et al., 2022). Table 1 demonstrates the characteristics of every included research and the quality scores of all cohort studies. The median (range) score of quality was 7.5 (Lawrance et al., 2017; Bao et al., 2019), and all three cohort studies were assessed as having high quality. The details of the quality assessment of the three cohort studies are given in Table 2. The bias evaluation plots of the four RCT studies are given in Figure 2. Figures 3, 5 show the forest plot and funnel plot for the comparison of clinical outcomes in the LCPT and PR-Tac groups, respectively, and Figures 4, 6 show those in the LCPT and IR-Tac groups, respectively.

**TABLE 1:** Baseline characteristics of included studies and the methodological assessment.

Author	Study period	Location	Study design	Patients (n)	Median follow-up (months)	Quality score
				LCPT/IR-Tac/PR-Tac		
Budde et al	2020–2021	Europe	RCT	200/86/115	6	NA
Glander et al	2017–2018	Germany	Cohort	23/23/36	12	7
Czarnecka et al	2016–2019	Poland	Cohort	59/0/56	24	8
Fernandez et al	2016–2017	Spain	Cohort	129/0/89	6	7
Kamar et al	2015–2016	France	RCT	33/0/36	1	NA
Rostaing et al	2010–2014	United States, Latin America, Europe, and Asia–Pacific	RCT	268/275/0	24	NA
Budde et al	2010–2013	United States, Latin America, Europe, and Asia–Pacific	RCT	268/275/0	12	NA

**TABLE 2:** Quality evaluation of the eligible studies with the Newcastle–Ottawa scale.

		Selection			Comparability			Outcome	
Study	Representativeness	Selection of non-exposed	Ascertainment of exposure	Outcome not present at the start	Comparability of the most important factors	Comparability of other risk factors	Assessment of outcomes	Long enough follow-up (median ≥ 1 year)	Adequacy (completeness) of the follow-up
Glander et al	^a^	^a^	^a^	^a^	^a^	—	^a^	^a^	—
Fernandez et al	—	^a^	^a^	^a^	^a^	^a^	^a^	—	^a^
Czarnecka et al	^a^	^a^	^a^	^a^	^a^	^a^	^a^	^a^	^a^

^a^ indicates the criterion met; — indicates significance of the criterion not met.

### 3.2 Demographic characteristics

Age (WMD: −0.14; 95% CI: −2.36, 0.07; *p* = 0.07), gender (male/total, OR: 0.99; 95% CI: 0.82, 1.19; *p* = 0.89), BMI (WMD: 0.31; 95% CI: −0.27, 0.89; *p* = 0.30), diabetes at the time of the transplant (OR: 0.99; 95% CI: 0.79, 1.24; *p* = 0.90), race (white/total, OR: 0.86; 95% CI: 0.66, 1.11; *p* = 0.24), donor type (living/total, OR: 1.14; 95% CI: 0.93, 1.40; *p* = 0.22), CIT (WMD: −0.68; 95% CI: −2.21, 0.85; *p* = 0.38), panel-reactive antibodies (WMD: −0.01; 95% CI: −0.66, 0.64; *p* = 0.97), and previous transplant (OR: 0.95; 95% CI: 0.54, 1.65; *p* = 0.84) did not present significant differences between groups. Then, we performed subgroup analysis for the demographic characteristics in the LCPT, PR-Tac, and IR-Tac groups, and the results showed no statistical significance (Table 3).

**TABLE 3:** Demographics and clinical characteristics of included studies.

Outcome	Studies	Number of patients	WMD or OR	95% CI	*p*-value	Heterogeneity			
						Chi^2^	df	*p*-value	*I* ^2^ (%)
**Age (years)**		**LCPT/IR-Tac + PR-Tac**							
	7	980/991	−1.14	[-2.36, 0.07]	0.07	8.20	6	0.22	*27*
		**LCPT/PR-Tac**							
	5	444/332	0.50	[-2.39, 3.39]	0.73	8.55	4	0.07	*53*
		**LCPT/IR-Tac**							
	3	491/384	−1.30	[-3.20, 0.60]	0.18	1.41	2	0.49	*0*
**Gender (male)**		**LCPT/IR-Tac + PR-Tac**							
	7	980/991	0.99	[0.82, 1.19]	0.89	3.68	6	0.72	0
		**LCPT/PR-Tac**							
	5	444/332	0.99	[0.72, 1.35]	0.95	3.64	4	0.46	0
		LCPT/IR-Tac							
	3	491/384	1.04	[0.78, 1.39]	0.78	0.62	2	0.73	0
**BMI (kg/m** ^**2**^ **)**		**LCPT/IR-Tac + PR-Tac**							
	5	444/441	0.31	[-0.27, 0.89]	0.30	4.51	4	0.34	11
		**LCPT/PR-Tac**							
	5	444/332	0.16	[-0.46, 0.78]	0.62	3.10	4	0.54	0
		**LCPT/IR-Tac**							
	2	223/109	0.51	[-1.27, 2.30]	0.57	2.11	1	0.15	53
**Diabetes at the time of transplant**		**LCPT/IR-Tac + PR-Tac**							
	6	945/955	0.99	[0.79, 1.24]	0.90	6.35	5	0.27	21
		**LCPT/PR-Tac**							
	4	411/296	1.08	[0.75, 1.57]	0.67	5.39	3	0.15	44
		**LCPT/IR-Tac**							
	3	491/384	0.99	[0.70, 1.41]	0.97	2.03	2	0.36	2
**Race (White people)**		**LCPT/IR-Tac + PR-Tac**							
	6	921/935	0.86	[0.66, 1.11]	0.24	5.16	4	0.27	22
		**LCPT/PR-Tac**							
	4	385/276	0.75	[0.21, 2.62]	0.65	6.12	2	0.05	67
		**LCPT/IR-Tac**							
	3	491/384	0.85	[0.58, 1.24]	0.40	1.09	2	0.58	0
**Donor type (living)**		**LCPT/IR-Tac + PR-Tac**							
	6	921/935	1.14	[0.93, 1.40]	0.22	0.10	4	1.00	0
		**LCPT/PR-Tac**							
	3	256/187	1.14	[0.70, 1.85]	0.60	0.07	2	0.97	0
		**LCPT/IR-Tac**							
	3	491/384	1.13	[0.85, 1.51]	0.41	0.88	2	0.64	0
**CIT(h)**		**LCPT/PR-Tac**							
	2	188/145	−0.68	[-2.21, 0.85]	0.38	0.09	1	0.76	0
**PRA**		**LCPT/IR-Tac + PR-Tac**							
	3	595/606	−0.01	[-0.66, 0.64]	0.97	0.05	2	0.97	0
**Previous transplant**		**LCPT/IR-Tac + PR-Tac**							
	4	592/645	0.95	[0.54, 1.65]	0.84	1.84	3	0.61	0
		**LCPT/PR-Tac**							
	2	56/72	0.61	[0.04, 9.91]	0.73	2.11	1	0.15	53
		**LCPT/IR-Tac**							
	2	291/298	1.02	[0.45, 2.32]	0.95	0.00	1	0.99	0

BMI, body mass index; CIT, cold ischemic time; PRA, panel-reactive antibody; WMD, weighted mean difference; OR, odds ratio; CI, confidence interval.

Italic value indicates that *p*-value > 0.05 prove demographics and clinical characteristics between the groups have no statistical significance. When *I* ^2^ > 50% was considered as evidence of heterogeneity in demographics and clinical characteristics between the groups.

### 3.3 PK profile

The PK profile was used in this study to represent relative bioavailability, which was expressed as the ratio of the blood concentration level to the tacrolimus dose (C/D). Pharmacokinetic data for comparing LCPT and PR-Tac and LCPT and IR-Tac were obtained from four and three studies, respectively. The four studies included 707 patients (411 for LCPT and 296 for PR-Tac), and the three studies included 875 patients (491 for LCPT and 384 for IR-Tac). The pooled analysis showed that the LCPT group had a higher PK profile than the PR-Tac (WMD: 0.74; 95% CI: 0.45, 1.04; *p* < 0.00001) (Figure 3A) and the IR-Tac groups (WMD: 0.48; 95% CI: 0.30, 0.67; *p* < 0.00001) (Figure 4A). There was significant heterogeneity only in the pooled analysis between the LCPT and PR-Tac groups (I^2^ = 67%, *p* = 0.03). The visual funnel plots for the publication bias assessment suggest no bias in publication (Figures 5A, 6A).

### 3.4 TTL

The TTL for comparing LCPT and PR-Tac and LCPT and IR-Tac was analyzed in four studies involving 558 patients (315 for LCPT and 243 for PR-Tac) and in two studies involving 332 patients (223 for LCPT and 109 for IR-Tac), respectively (Glander et al., 2018; Kamar et al., 2019; Budde et al., 2022; Czarnecka et al., 2023). According to the pooled analysis, TTL was similar between the LCPT and PR-Tac groups (WMD: 0.33; 95% CI: −0.42, 1.08; *p* = 0.39) (Figure 3B) and the LCPT and IR-Tac groups (WMD: 0.06; 95% CI: -0.51, 0.63; *p* = 0.83) (Figure 4B), and heterogeneity was substantial only in the analysis of LCPT and PR-Tac (I^2^ = 64; *p* = 0.04). The funnel plots show no publication bias (Figures 5B, 6B).

### 3.5 Changes in eGFR

Two articles reported changes in eGFR, containing 184 patients (92 for LCPT and 92 for PR-Tac) (Kamar et al., 2019; Czarnecka et al., 2023). There was no substantial difference in the change in eGFR between both groups (SMD: 0.02; 95% CI: −0.27, 0.31; *p* = 0.90) (Figure 3C), with no substantial heterogeneity (I^2^ = 0%; *p* = 0.75) and no publication bias. A visual funnel plot for the publication bias assessment shows no bias in publication (Figure 5C).

### 3.6 Changes in Scr

Two articles reported changes in Scr, containing 184 patients (92 for LCPT and 92 for PR-Tac) (Kamar et al., 2019; Czarnecka et al., 2023). There was no substantial difference in the change in eGFR between both groups (WMD: 0.33; 95% CI: −1.09, 0.42; *p* = 0.39) (Figure 3D), with no substantial heterogeneity (I^2^ = 0%, *p* = 0.66). A visual funnel plot for the publication bias assessment shows no bias in publication (Figure 5D).

### 3.7 BPAR rate

BPAR rates for comparing LCPT and PR-Tac, and LCPT and IR-Tac were obtained from 459 patients (242 for LCPT and 217 for PR-Tac) in four studies and 587 patients in two studies (289 for LCPT and 298 for IR-Tac), respectively (Rostaing et al., 2016; Glander et al., 2018; Kamar et al., 2019; Fernandez Rivera et al., 2022; Czarnecka et al., 2023). There was no significant difference between the LCPT and PR-Tac groups (OR: 0.90; 95% CI: 0.48, 1.70; *p* = 0.76) (Figure 3E) or the LCPT and IR-Tac groups (OR: 0.91; 95% CI: 0.59, 1.38; *p* = 0.64) (Figure 4C), and no significant heterogeneity was found for both comparisons (I^2^ = 0%, *p* = 0.60; I^2^ = 0%, *p* = 0.66). The funnel plots indicate no significant publication bias (Figures 5E, 6C).

### 3.8 DGF rate

Four studies analyzed the DGF rate for comparing between LCPT and PR-Tac, involving 459 patients (242 for LCPT and 217 for PR-Tac), and two studies analyzed the DGF rate for comparing between LCPT and IR-Tac, including 587 patients (289 for LCPT and 298 for IR-Tac) (Budde et al., 2014; Glander et al., 2018; Kamar et al., 2019; Fernandez Rivera et al., 2022; Czarnecka et al., 2023). The pooled analysis suggested no significant difference between the LCPT and PR-Tac groups (OR: 0.90; 95% CI: 0.57, 1.44; *p* = 0.67) (Figure 3F) or the LCPT and IR-Tac groups (OR: 0.75; 95% CI: 0.43, 1.28; *p* = 0.29) (Figure 4D). No substantial heterogeneity (I^2^ = 0%, *p* = 0.84; I^2^ = 50%, *p* = 0.16) or visible evidence of publication bias was observed (Figures 5F, 6D).

### 3.9 PTDM rate

Three articles reported the PTDM rate, involving 402 patients (221 for LCPT and 181 for PR-Tac) (Kamar et al., 2019; Fernandez Rivera et al., 2022; Czarnecka et al., 2023). No considerable difference (OR: 0.80; 95% CI: 0.48, 1.35; *p* = 0.40) (Figure 3G) or statistically noticeable heterogeneity (I^2^ = 0%, *p* = 0.45) was found in the two groups. Simultaneously, the funnel plot shows no publication bias (Figure 5G).

### 3.10 TR

Two studies reported TR, involving 287 patients (162 for LCPT and 125 for PR-Tac) (Kamar et al., 2019; Fernandez Rivera et al., 2022). Evidence synthesis revealed a similar TR in both groups (OR: 0.54; 95% CI: 0.23, 1.31; *p* = 0.17), with no remarkable heterogeneity (I^2^ = 29%, *p* = 0.24) (Figure 3H), and no visible evidence of publication bias was observed (Figure 5H).

### 3.11 DR

DR was obtained from 333 patients (188 for LCPT and 145 for PR-Tac) in four studies (Fernandez Rivera et al., 2022; Czarnecka et al., 2023). The pooled analysis revealed an equal DR between both groups (OR: 0.39; 95% CI: 0.07, 2.09; *p* = 0.27) (Figure 3I). No remarkable heterogeneity (I^2^ = 0%, *p* = 0.43) or visible evidence for publication bias was found (Figure 5I).

### 3.12 Rate of infection by CMV

Two articles reported the rate of infection by CMV, involving 295 patients (167 for LCPT and 128 for PR-Tac) (Fernandez Rivera et al., 2022; Czarnecka et al., 2023). There was no significant difference between the LCPT and PR-Tac groups (OR: 0.87; 95% CI: 0.50, 1.52; *p* = 0.62) (Figure 3J). No remarkable heterogeneity (I^2^ = 41%, *p* = 0.19) or visible evidence for publication bias was found (Figure 5J).

### 3.13 Sensitivity analysis

**FIGURE 7:**
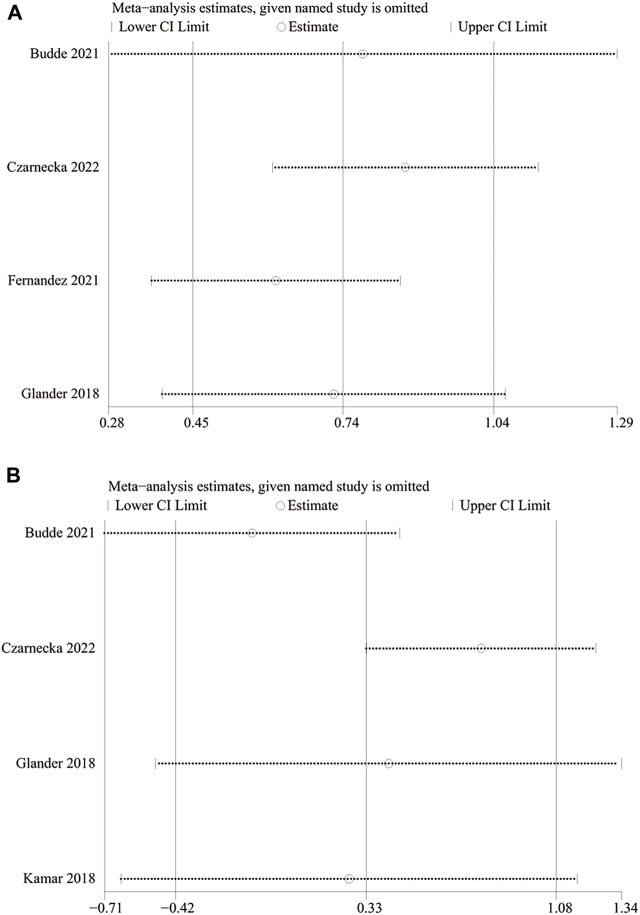
Sensitivity analysis of **(A)** PK (LCPT versus PR-Tac) and **(B)** TTL (LCPT versus PR-Tac).

We carried out a one-way sensitivity analysis by excluding individual studies one by one to assess the effect of each study on WMD of the combined study. Sensitivity analysis showed that the pooled WMD remained unchanged after exclusion of studies one by one for the PK profile (LCPT versus PR-Tac) (Figure 7A). However, the heterogeneity regarding the PK profile disappeared (I^2^ = 43%, *p* = 0.17; I^2^ = 31%, *p* = 0.23) after the studies by Fernandez et al. reported in 2021 and Czarnecka et al. reported in 2022 were removed separately, demonstrating that these two studies explained the sources of heterogeneity. In the sensitivity analysis of TTL for LCPT versus PR-Tac, the WMD changed after the study by Budde et al. was removed (Budde et al., 2022), but the heterogeneity disappeared (I^2^ = 0%, *p* = 0.60). When the study by Czarnecka et al. was removed (Czarnecka et al., 2023), WMD did not change, but the results became statistically significant and the heterogeneity disappeared (I^2^ = 2%, *p* = 0.36) (Figure 7B).

## 4 Discussion

Our work compared the PK profile and other outcomes in *de novo* KTRs receiving novel once-daily extended-release tacrolimus (LCPT) or standard-of-care tacrolimus (PR-Tac/IR-Tac). A systematic literature search was conducted for all eligible studies that were published from the inception of the databases up to 6 March 2023. Seven studies containing a total of 1,428 KTRs were included, based on a complete, systematic, and up-to-date review of the literature. Some data, such as changes in eGFR and Scr, PTDM rate, TR, DR, and the rate of infection by CMV, were reported in only one or two studies comparing LCPT and IR-Tac. Therefore, we only analyzed the comparison between LCPT and PR-Tac. The pooled analysis showed a significantly higher PK profile in the LCPT group than in the PR-Tac group (WMD: 0.74; 95% CI: 0.45, 1.04; *p* < 0.00001) and IR-Tac group (WMD: 0.48; 95% CI: 0.30, 0.67; *p* < 0.00001). There was a significant heterogeneity for LCPT versus PR-Tac (I^2^ = 67%, *p* = 0.03), and the heterogeneity was not significant for LCPT versus IR-Tac (I^2^ = 30%, *p* = 0.24). All other observed indicators were compared between LCPT and PR-Tac, and LCPT and IR-Tac.

The results of this meta-analysis for the PK profile were consistent with those of the seven included articles, providing strong evidence that the relative bioavailability of LCPT is superior to that of IR-Tac/PR-Tac. The evaluation of the PK profile between the LCPT and the PR-Tac groups showed that heterogeneity disappeared after either the study by Fernandez et al., in 2021 or the study by Czarnecka et al., in 2022 was eliminated, making the results robust, and the final results were still statistically significant. The pooled analysis of the TTL showed no significant difference between Envarsus and Prograf/Advagraf, and the results were consistent with those of three of the included studies and contrary to one of the included studies. Possible reasons were as follows: 1) the doses were adjusted to reach prespecified target levels in the clinical trial; 2) a limited number of patients in the included studies; 3) a limited number of relevant studies; and 4) the length of the time on medication of each study varies, resulting in the TTLs analyzed in this study being at different time points, which, in turn, may be influenced by the duration on medication. It is worth noting that a conclusion contrary to the pooled results was made, with no statistical significance, when the study by Budde et al. in 2021 was removed for the evaluation of TTL between the LCPT and PR-Tac groups. In contrast, the heterogeneity disappeared, and WMD did not change, making the conclusion that the LCPT group had statistically significant higher TTL, when the study by Czarnecka et al. in 2022 was removed. The main reason was that the abovementioned two studies made up a large proportion in the initial pooled analysis, and the conclusion obtained by Budde et al. was consistent with that of the pooled analysis, while the conclusion obtained by Czarnecka et al. was contrary to that of the pooled analysis. Therefore, the findings about TTL between LCPT and PR-Tac should be interpreted with caution, and more relevant studies should be conducted.

To investigate the differences between different groups in the effects of tacrolimus preparations on kidney function, this study evaluated various parameters for kidney function, including changes in eGFR and Scr. eGFR is generally considered the optimum indicator for kidney graft function and also the prediction of long-term transplantation and recipient survival (Inker et al., 2018). Kamar et al. indicated that the PR-Tac group had higher eGFR elevation than the LCPT group (Kamar et al., 2019), while Czarnecka et al. showed that the LCPT group had higher eGFR elevation than the PR-Tac group, but these findings were not statistically important. Our result was consistent with that obtained by Czarnecka et al., with no statistical significance. The result on the changes in Scr in our study was consistent with those of the two included studies, and all showed that the LCPT group had higher Scr reduction, although the results were not statistically significant. Therefore, we believe that the effects of LCPT and PR-Tac on the changes of eGFR and Scr need to be further discussed, and more relevant studies are needed to prove which agent is more helpful in improving the renal function. Of the included studies, one reported the incidence of BPAR 1 month later, two reported the incidence of BPAR 6 months later, two reported the incidence of BPAR after 1 year, and the remaining two reported the incidence of BPAR after 2 years. Pooling the incidence of BPAR across all studies, we observed no substantial differences between both groups regarding this outcome, and this was in line with the results of a previous meta-analysis (Saengram et al., 2018). In addition, no statistically significant differences were found between LCPT and PR-Tac or between LCPT and IR-Tac for the DGF rate, PTDM rate, TR, adverse event rate, GFR, DR, and the rate of infection by CMV, which are indicators of interest to clinicians.

This study had limitations that were inherent to meta-analysis, especially for kidney transplantation outcomes. First, the included studies were small in number and sample sizes. Second, although trials registered in various databases were searched and publications in English and in all other languages were considered, reporting bias could not be completely excluded. Third, there may be publication bias due to overestimated or underestimated results. Finally, all of our findings are objective, but some studies were not blinded or failed to provide relevant information about random sequence generation and/or allocation concealment, which might lead to performance bias and selection bias.

## 5 Conclusion

The pooled analysis showed that tablet formulation of tacrolimus had a PK profile advantage over capsule formulation of tacrolimus in *de novo* KTRs. Given the heterogeneity between studies and potential bias, the care providers should choose a dosing strategy in accordance with the specific circumstances of patients and their clinical experience for the immunosuppressive treatment of *de novo* KTRs.

## Data Availability

The original contributions presented in the study are included in the article/Supplementary Material; further inquiries can be directed to the corresponding author.
